# Unresectable Intermediate-Size (3–5 cm) Colorectal Liver Metastases: Stereotactic Ablative Body Radiotherapy Versus Microwave Ablation (COLLISION-XL): Protocol of a Phase II/III Multicentre Randomized Controlled Trial

**DOI:** 10.1007/s00270-023-03498-8

**Published:** 2023-07-10

**Authors:** Susan van der Lei, Madelon Dijkstra, Sanne Nieuwenhuizen, Hannah H. Schulz, Danielle J. W. Vos, Kathelijn S. Versteeg, Tineke E. Buffart, Rutger-Jan Swijnenburg, Jan J. J. de Vries, Anna M. E. Bruynzeel, M. Petrousjka van den Tol, Hester J. Scheffer, Robbert S. Puijk, Cornelis J. A. Haasbeek, Martijn R. Meijerink, and COLLISION Trial Group, Bart Geboers, Floor E. F. Timmer, Henk Verheul, Karin Nielsen, Bram Van der Meijs, Nicole Van Grieken, Otto Van Delden, Thomas Van Gulik, Mark Besselink, Pieter Tanis, Krijn Van Lienden, Mark Burgmans, Arian Van Erkel, Henk Hartgrink, Carla Van Rijswijk, Sven Mieog, Colin Sietses, Tjarda Van Heek, Arjen Diederik, Gert-Jan Spaargaren, Gerie Groot, Ted Vink, Eric Manusama, Hasan Eker, Johan Dol, Ingrid Kappers, Christiaan Van der Leij, Rutger Brans, Mariëlle Coolsen, Kees De Jong, Ronald Van Dam, Han Kruimer, Laurens Van Baardewijk, Wouter Leclercq, Jurgen Futterer, Peter Van den Boezem, Martijn Stommel, Hans De Wilt, Sjoerd Jenniskens, Mark Arntz, Jan Jaap Janssen, Hans Torrenga, Simeon Ruiter, Maarten Nijkamp, Matthijs Kater, Koert De Jong, GianPiero Serafino, Werner Draaisma, Anne Van Geel, Hermien Schreurs, Maarten Smits, Jeroen Hagendoorn, Quintus Molenaar, Rutger Bruijnen, Warner Prevoo, Francesco De Cobelli, Luca Aldrighetti, Francesca Ratti, Paolo Marra, Angelo Della Corte, Thiery Chapelle, Marc Peeters

**Affiliations:** 1 Department of Radiology and Nuclear Medicine, Amsterdam UMC, Location VUmc, De Boelelaan 1117, 1081 HV Amsterdam, The Netherlands; 2 Department of Medical Oncology, Amsterdam UMC, Location VUmc, Amsterdam, The Netherlands; 3 Department of Surgery, Amsterdam UMC, Location VUmc, Amsterdam, The Netherlands; 4 Department of Radiation Oncology, Amsterdam UMC, Location VUmc, Amsterdam, The Netherlands; 5 Department of Surgery, Medical Center Leeuwarden, Leeuwarden, The Netherlands; 6 Department of Radiology and Nuclear Medicine, OLVG Hospital, Amsterdam, The Netherlands; 7 Department of Radiology and Nuclear Medicine, NWZ Hospital Group, Alkmaar, The Netherlands

**Keywords:** Colorectal cancer, Colorectal liver metastases (CRLM), Liver metastases, Thermal ablation, Microwave ablation (MWA), Radiotherapy, Stereotactic body radiotherapy (SBRT), Randomized controlled trial (RCT)

## Abstract

**Background:**

Although microwave ablation (MWA) has a low complication rate and good efficacy for small-size (≤ 3 cm) colorectal liver metastases (CRLM), local control decreases with increasing size. Stereotactic body radiotherapy (SBRT) is gaining interest as a potential means to treat intermediate-size CRLM and might be less susceptible to increasing volume. The objective of this study is to compare the efficacy of MWA to SBRT in patients with unresectable, intermediate-size (3–5 cm) CRLM.

**Methods:**

In this two-arm, multicentre phase II/ III randomized controlled trial, 68 patients with 1–3 unresectable, intermediate-size CRLM suitable for both MWA and SBRT, will be included. Patients will be treated with MWA or SBRT as randomised. The Primary endpoint is local tumour progression-free survival (LTPFS) at 1 year (intention-to-treat analysis). Main secondary endpoints are overall survival, overall and distant progression-free survival (DPFS), local control (LC) and procedural morbidity and mortality and assessment of pain and quality of life.

**Discussion:**

Current guidelines lack clear recommendations for the local treatment of liver only intermediate-size, unresectable CRLM and studies comparing curative intent SBRT and thermal ablation are scarce. Although safety and feasibility to eradicate tumours ≤ 5 cm have been established, both techniques suffer from lower LTPFS and LC rates for larger-size tumours. For the treatment of unresectable intermediate-size CRLM clinical equipoise has been reached. We have designed a two-armed phase II/ III randomized controlled trial directly comparing SBRT to MWA for unresectable CRLM 3–5 cm.

***Level of Evidence*:**

Level 1, phase II/ III Randomized controlled trial.

*Trial Registration*: NCT04081168, September 9th 2019.

**Graphical Abstract:**

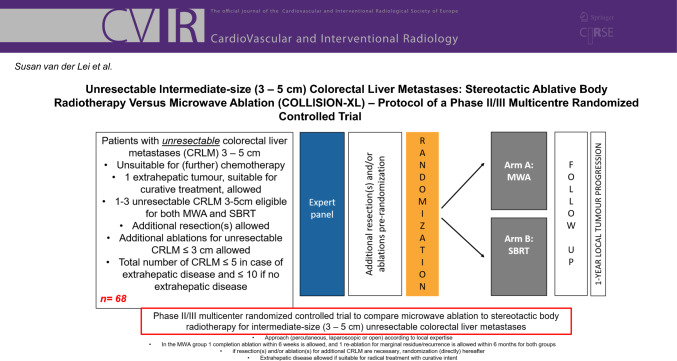

## Introduction

Colorectal carcinoma is one of the most common malignancies, with 1.8 million new cases worldwide in 2018 [1]. In the course of the disease roughly 30–50% of patients develop colorectal liver metastases (CRLM) [2, 3]. Although chemotherapeutic regimens are improving, local therapy remains the only option associated with a realistic chance of long-term disease control or in selected cases even cure. Surgical resection represents the historical standard and treatment of first choice with 5-year overall survival (OS) reaching 40–55% [5, 6]. However, only a minority of the patients (20–30%) is eligible for surgery, due to insufficient future liver remnant after resection, unfavourable anatomical location of the metastases, concomitant co-morbidities or patients with previous extensive abdominal surgery [7]. For the eradication of liver only and limited unresectable metastases, several ablative strategies have emerged.

Thermal ablation (i.e., radiofrequency ablation (RFA), microwave ablation (MWA)) for small liver tumours has an excellent safety profile with a low complication rate and it offers good local control. Local tumour progression (LTP) rates range 0–10% after thermal ablation for tumours ≤ 3 cm [8–12]. However, in tumours > 3 cm the efficacy rapidly decreases, with LTP rates ranging 14–45% [8, 9, 13]. For these larger-size tumours, novel MWA generation systems or tumour-bracketing multi-probe ablation techniques are preferred. Stereotactic body radiotherapy (SBRT) is gaining more interest as a potential treatment method for intermediate-size CRLM. A recent propensity score-based comparison favoured SBRT over thermal ablation for CRLM > 3 cm regarding local control after initial treatment [16]. Yet it seems unjust to compare local control rates following only one ablative procedure, of a one-shot treatment method (SBRT), with an easily repeatable technique (MWA).

This multi-centre phase-II/ III randomized controlled trial was designed to compare the efficacy of SBRT versus MWA in unresectable intermediate-size (3–5 cm) CRLM. The aim is to prove superiority of SBRT or MWA regarding to 1 year local tumour progression free survival.

## Design

### Design

**Fig. 1:**
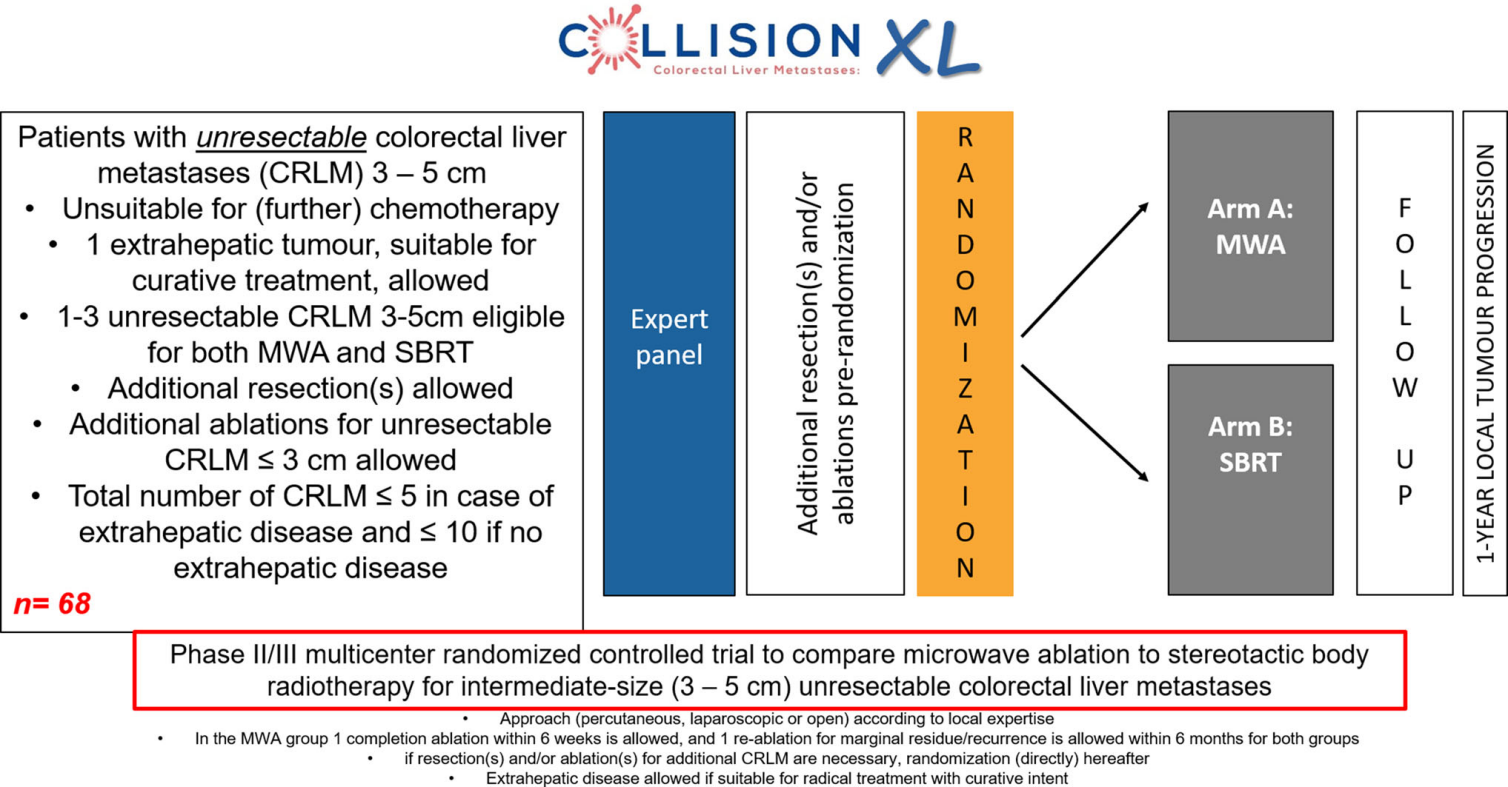
Flowchart of the study design

The COLLISION-XL trial is a phase II/III, multicentre randomized controlled trial initiated by the Amsterdam University Medical Centres (Amsterdam UMC, location VUmc in Amsterdam, the Netherlands) and endorsed by the Dutch Colorectal Cancer Group (DCCG). Patients will be recruited in at least five hospitals in the Netherlands: Amsterdam UMC (location VUmc), Amsterdam; Ziekenhuis Gelderse Vallei (ZGV), Ede; University Medical Centre Groningen (UMCG), Groningen; Noordwest hospitals, Alkmaar; Medical centre Leeuwarden, Leeuwarden. The trial is investigator-initiated and funded by a partially restricted grant from Johnson & Johnson and registered at clinicaltrials.gov under NCT04081168. The protocol has been approved by the medical Ethical Review Board (METc) of the Amsterdam UMC, location VUmc (no. 2019.262–NL68326.029.19). The trial will be conducted in accordance with the Declaration of Helsinki and the guidelines for Good Clinical Practice (GCP). The duration of the study will be around 4 years (3 years for inclusion and 1 year follow-up). The flow chart of the study design is shown in Fig. 1.

### Inclusion Criteria

Patients with 1–3 unresectable intermediate-size CRLM, suitable for both MWA and SBRT are eligible to participate. An overview of inclusion and exclusion criteria is listed in Table 1. Additional CRLM are allowed if they are either resectable or < 3 cm and amenable for thermal ablation. In total, a maximum of 10 CRLM without extrahepatic disease or 5 CRLM in case of limited extrahepatic disease is allowed. Prior focal liver treatment is allowed, but resection or ablation site recurrences are disqualified as target lesions. Patients without prior focal liver treatment should be either unsuitable for 1st line systemic therapy or have progressed under/after 1st-line systemic therapy. Potential candidates will be registered and undergo routine pre-procedural work-up: baseline full blood examination, ceCT of the chest and abdomen and either an upper abdominal ceMRI or a total body ^18^F-FDG PET-CT.

**Table 1:** Inclusion and exclusion criteria

Inclusion criteria	Exclusion criteria
Histological documentation of primary colorectal tumour	No target lesions suitable for both ablation and SBRT
Age > 18 years	Chemotherapy ≤ 6 weeks prior to the procedure
1–3 unresectable CRLM size 3–5 cm eligible for both MWA and SBRT (target lesions)	Immunotherapy ≤ 6 weeks prior to the procedure
Additional CRLM are allowed if considered either resectable, or ablatable and < 3 cm	Severe allergy to contrast media not controlled with premedication
No or limited extrahepatic disease (1 extrahepatic lesion is allowed, not including positive para-aortal lymph nodes, celiac lymph nodes, adrenal metastases, pleural carcinomatosis or peritoneal carcinomatosis)	Compromised liver function (e.g. signs of portal hypertension, INR > 1,5 without use of anticoagulants, ascites)
For subjects with liver only disease the maximum number of CRLM is 10; for subjects with limited extrahepatic disease the maximum number of CRLM is 5	Radical treatment unfeasible or unsafe (e.g. insufficient future liver remnant [FLR])
Resection for additional resectable lesions is allowed when considered possible obtaining negative resection margins (R0) and preserving adequate future liver remnant	Pregnant or breast-feeding subjects
Prior focal liver treatment is allowed, but resection or ablation site recurrences are disqualified as target lesions	
Unsuitable for (further) systemic therapy	
Written informed consent	

Participants will be stratified according to presence of extrahepatic disease and whether patients will undergo surgical resection. An experienced expert panel, consisting of at least two interventional radiologists, two hepatobiliary surgeons and two radiation oncologists, will determine whether the CRLM are (un)resectable and/or (un)ablatable. Unresectable intermediate-size CRLM suitable for both MWA and SBRT are so-called target-tumours. All additional non-target tumours should be resectable or, if unresectable and ≤ 3 cm, eligible for thermal ablation.

### Exclusion Criteria

Patients are considered ineligible and will not be randomized pre-procedurally or during surgical inspection or IOUS if (1) the maximum amount of CRLM is exceeded, (2) there are no target tumours, (3) impermissible extrahepatic disease is present or (4) radical treatment is no longer feasible. Pre-procedurally appointed target tumours that prior to randomization prove to be suitable for resection or unsuitable for either MWA or SBRT during surgery, are no longer regarded as target tumours, potentially excluding patients from further study participation.

### Statistics

Given the unknown 1 year LTP rate for intermediate-size CRLM for both techniques, it was not possible to apply validated assumptions for both proportions. Hence a difference in 1y-LTP rate of more than one third (33%) is considered clinically relevant as it seems unlikely that a smaller difference will significantly impact overall survival. Most 1-year LTP will either be re-ablated or patients will receive systemic chemotherapy because of the simultaneous presence of extensive distant progression. In other words, unablatable LTP at 1-year without recurrence at distant sites is uncommon. We therefore accept that a difference in 1 year LTPFS of more than one third (0.33) will reject the null hypothesis concluding that the techniques are significantly different [17]. Group sample sizes of 33 in group 1 and 33 in group 2 achieve 81.059% power to detect a difference between the group proportions of −0.33. The proportion in group 1 (the treatment group) is assumed to be 0.665 under the null hypothesis and 0.3350 under the alternative hypothesis. The proportion in group 2 (the alternative treatment group) is 0.665. The test statistic used is the two-sided Z-test with un-pooled variance. The significance level of the test is 0.05. To account for a 3% loss to follow-up (= 2 patients) we need to randomize 34 patients (NR) into each of the two arms.

Baseline patient-, tumour- and treatment characteristics will be described and compared between the two treatment arms using the Chi-square test for categorical variables and the independent two-sample t-test, ANOVA or Mann–Whitney-U-test for continuous variables. *P* values below 0.05 will be considered significant. When appropriate, box-plots and cross-tables will be used for descriptive statistics of continuous and categorical variables, respectively. One year LTPFS rates will be compared between MWA and SBRT using the Chi square test. To determine hazard ratios (HR) for multivariate analysis, logistic regression will be used. Univariate LTPFS time analysis will be performed using the Kaplan–Meier method with corresponding two sided 95% confidence intervals (CI). Differences in LTPFS length will be analysed using the log rank test.

### Study Cohort

Patients eligible for percutaneous ablation will be randomized prior to the procedure into one of two study arms: percutaneous MWA (arm A) or SBRT (arm B). Patients with at least one additional CRLM requiring resection will undergo laparoscopy or laparotomy with surgical inspection of the abdominal cavity and intraoperative ultrasound (IOUS) and if still considered eligible randomized intraoperatively. If randomized to MWA during open laparotomy, the eligible tumours will be ablated in the same procedure using ultrasound guidance. Patients included in study arm B will undergo SBRT at a later time. SBRT will be planned as soon as the patient is recovered from surgery. Patients randomized prior to treatment will have to be treated within 4 weeks after randomization.

### Randomization

Blocked randomization with randomly selected block sizes of 4 and 6 will be performed centrally through a web-based module (CASTOR), with stratification according to presence of extrahepatic disease and patients undergoing surgical resection. According to the intention-to-treat concept, during statistical analysis all randomized patients will be analysed in the group they were originally assigned to even when receiving a different treatment.

### Microwave Ablation

MWA procedures will be performed by a board certified operator striving for a tumour free ablation margin of at least 1 cm, according to the CIRSE quality improvement guidelines [18]. Contra -indications for a percutaneous approach are proximity of critical structures that cannot be distanced using pneumo- or hydrodissections. The interventional radiologist will assess the necessity for overlapping re-ablations and needle repositioning. To assess technical efficacy a ceCT (or ceMRI) should be made within two weeks after the procedure. Completion ablations for residual disease or inadequate tumour-free safety margins are allowed if detected on cross-sectional imaging and retreated ≤ 6 weeks after initial treatment. In this case, the initial procedure will be evaluated as a technically unsuccessful procedure. However, when successfully re-ablated the retreated residue will not count as an event for the primary endpoint LTPFS. If successfully re-ablated within 6 months after the initial treatment, early ablation site recurrence will not be regarded as an event for the primary endpoint 1 year LTPFS. The use of either rigid and/or non-rigid confirmation software is considered mandatory aiming at a minimum of 5 mm margins.

### Stereotactic Body Radiotherapy

SBRT will be delivered in an image-guided hypofractionated scheme of 60 Gy in 3, 5 or 8 fractions, prescribed to 95% of the planning target volume (PTV). The 5 or 8 fraction schedule will be used in case of overlap with central hilar structures, large vessels, gallbladder or in cases where under-dosing the gross tumour volume (GTV)/PTV is necessary due to near critical structures (oesophagus, stomach, duodenum, bowel). In preparation for treatment delivery, all patients will undergo a planning-CT scan or a planning MRI in case of MR guided treatment. Abdominal compression, gated treatment or breath hold may be used, but is not mandatory. Intravenous contrast may be used during simulation; however, it is not required. Fusion will be centred on the area of interest i.e. the CRLM. The PTV will be generated with the addition of a 3–10 mm margin around the gross tumour volume (GTV) depending on institutional protocols. The duodenum, stomach, bowel, liver, kidneys, and spinal cord will be contoured as avoidance structures. Other tissue structures will be contoured depending on the method of motion management. When an internal target volume (ITV) concept is used including motion, motion should also be included in the delineation of critical structures for maximum critical dose (i.e. oesophagus, stomach, bowel, duodenum). The liver should be delineated on the average phase bin and not include all motion, otherwise the volume of low liver dose will be overestimated. Radiation therapy will be delivered using any high-precision SBRT technique; this can be in the form of MR guided radiotherapy, intensity-modulated radiotherapy (IMRT), volumetric modulated arc therapy (VMAT), tomotherapy, or using multiple non-coplanar fields or arcs. If disease recurrence is detected and treated with re-SBRT within 6 months after the initial treatment the radiation-site recurrence will not be regarded as an event for the primary endpoint. Re-SBRT will have to be performed with sufficiently high doses to be considered ablative.

### Follow-Up

**Fig. 2:**
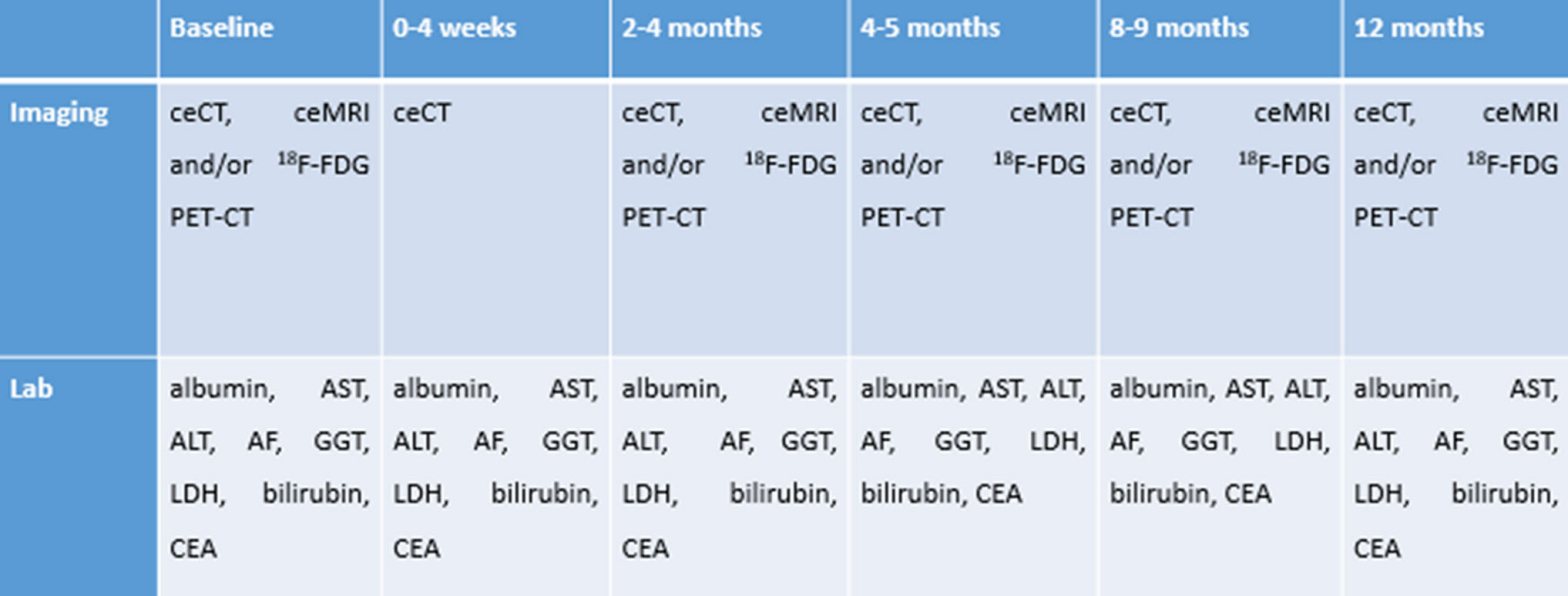
Follow-up

Follow-up imaging and laboratory tests will be identical in both treatment arms to avoid bias with regards to both the primary and secondary endpoints such as distant progression (Fig. 2). Patients will be asked to complete quality of life questionnaires at baseline and every 3 months for the first year. All potential LTPs will be reviewed by an expert panel including independent abdominal radiologists in presence of the study PI’s.

### Outcome Measures

The primary objective is to compare 1-year local tumour progression free survival (LTPFS) from randomisation per treated tumour in both study arms. The 1 year LTPFS is defined as the percentage of successfully eradicated tumours 12 months following treatment, according to the RECIST and PERCIST criteria with a standardised follow-up protocol. LTP successfully retreated within the first 6 months, using the same technique as the initial treatment (re-MWA or re-SBRT) will not be regarded as an event for the primary endpoint. Secondary endpoints are: OS, local control (allowing repeat treatments with the same technique) overall and distant progression-free survival (DPFS), rate of adverse events and serious adverse events, quality of life assessments using EORCT QLQ-C30, EQ-5D, and PRODISQ questionnaires prior to, and 2 weeks, 3, 6, 9 and 12 months after local treatment.

### Data Monitoring

The Clinical Research Bureau (CRB), an independent monitor committee, will safeguard the quality of this investigator-initiated study. An employee from the CRB will check the data according to Good Clinical Practice (GCP). Source Data verification will be performed during onsite monitoring (to verify if all data on the Case Report Form are in accordance with the source data). For all subjects, the informed consent forms, the inclusion and exclusion criteria and the primary outcome (1 year LTFPS) will be verified. The monitor will also verify if all (S) AE’s are reported adequately..

### Serious Adverse Events

All serious adverse events (SAE) occurring in the first 90 days after treatment, both related and unrelated will be reported within 15 days after the responsible investigator has first been notified of the SAE and within 7 days if an SAE is life-threatening or results in death. After these 90 days, only SAE’s related to the research according to one of the principal investigators, will be reported. SAEs will be reported through the web portal ToetsingOnline to the METc that approved the protocol.

## Discussion

Currently, guidelines state that thermal ablation can be applied for unresectable CRLM of limited-size (≤ 3 cm) and that SBRT should be considered if the location of the tumour is unfavourable for thermal ablation [19–21]. However, the preferred treatment option for intermediate-size unresectable CRLM (3-5 cm) has not been established. Following both thermal ablation and SBRT, LTP rates increase with larger tumour size [17, 22–28]. Patients eligible for both treatment options often receive extensive chemotherapy in an attempt to downstage patients from unresectable disease to resectable disease [20]. If tumours remain unresectable, thermal ablation and SBRT are a ‘last resort’ local curative intent treatment option with a presumed superior oncological outcome [3, 20].

The current primary efficacy rate of thermal ablation (complete ablation after the first procedure) for CRLM ≤ 3 cm (92–100%) is approaching to local recurrence rates after surgery for similar sized tumours [11, 29–32]. However, multiple studies have shown that efficacy significantly decreases to below 80% following thermal ablation of CRLM > 3 cm [11, 24, 33, 34]. For these larger-size tumours, novel MWA generation systems or tumour-bracketing multiprobe ablation techniques are preferred.As wide peri-ablational margins with > 5 mm margins, and if possible > 10 mm, are associated with technical success (A0 ablations), multiple electrodes may be used to increase the size of the ablation zone [35]. With the use of MWA, higher intra-tumoural temperatures, faster heating, shorter procedure times and larger ablation volumes with wider margins can be achieved, even in larger tumours [36–38]. Also, due to its active heating mechanism MWA might be less susceptible to the “heat-sink” effect of conductive heating with RFA [37].

The 1- and 2-year local control rate for SBRT in multiple studies (all retrospective or prospective cohort studies) ranges from 50 to 95% and 45–92%, respectively [39–49]. Several studies showed that increased tumour size had a negative influence on the local control rate [43, 44, 50]. Even with increased tumour sizes, SBRT can be administered safely when a tumour is located in proximity to the gallbladder, bile ducts, main vessels and the diaphragm. No anaesthesia is required and recent advances in SBRT techniques seem to have reduced radiation induced toxicity. With this new magnetic resonance (MR)-guidance technique, the tumour is visualized during radiation delivery. Radiation can be delivered “gated” (i.e. beam-on only when the tumour is in the predetermined position) using small uncertainty margins and thereby limiting the dose delivered to normal organs, likely resulting in decreased toxicity [51, 52]. Nonetheless, major complications still occur in 0–9% and toxicity grade I-II occurs in 23–78% of patients [43, 50, 53–56].

Studies directly comparing thermal ablation to SBRT for intermediate-size CRLM are scarce. One recent study compared thermal ablation to SBRT for all sizes of unresectable CRLM, where results should be interpreted with caution, because of potential residual confounding due to non-randomized nature and selection bias [57]. The LTPFS and LC significantly improved in the thermal ablation group compared to the SBRT group for all sizes. In addition, stratification of results of intermediate size CRLM significantly favoured thermal ablation over SBRT. In this study, size of CRLM was a significant predictor for development of LTP in the thermal ablation group. Nonetheless, size of CRLM did not predict the development of LTP in the SBRT group. Therefore, it was suggested that SBRT might be less susceptible to larger tumour-sizes. In contrast, a recent propensity score-based comparison of LC favoured SBRT over thermal ablation for intermediate size CRLM [16]. We chose to allow for one re-ablation for local tumour progression if detected within 6 months. It would otherwise be unjust to compare an easily repeatable ablative method (MWA) to a treatment that is more difficult to repeat safely (SBRT).

To conclude, as current guidelines lack clear recommendations for treatment of intermediate-size CRLM and as studies comparing the potential treatment options, SBRT and thermal ablation, are scarce, studies directly comparing the two treatment options are indispensable. COLLISION-XL represents a two-arm phase-II/ III randomized controlled trial directly comparing SBRT to MWA for unresectable, intermediate size CRLM.

## Abbreviations

^18^F-FDG: [18F]-fluoro-2-deoxy-D-glucose
COLLISION XL: Unresectable colorectal liver metastases: stereotactic body radiotherapy versus microwave ablation
Ce: Contrast-enhancement
CI: Confidence interval
CRC: Colorectal cancer
CRLM: Colorectal liver metastases
CT: Computed tomography
DPFS: Distant progression-free survival
HR: Hazard ratio
IRE: Irreversible electroporation
LC: Local tumour control
LTP: Local tumour progression
LTPFS: Local tumour progression-free survival
MRI: Magnetic resonance imaging
MWA: Microwave ablation
OS: Overall survival
PET: Positron emission tomography
RCT: Randomized controlled trial
RFA: Radiofrequency ablation
SBRT: Stereotactic body radiotherapy

## Declarations

### Human and Animal Participants

All procedures performed in studies involving human participants will be in accordance with the ethical standards of the institutional and/or national research committee and with the 1964 Helsinki declaration and its later amendments or comparable ethical standards.
